# Context is all you need: A causal framework for governing bias in clinical AI

**DOI:** 10.1371/journal.pdig.0001819

**Published:** 2026-11-06

**Authors:** Alon Gorenshtein, Mahmud Omar, Yiftach Barash, Girish N. Nadkarni, Eyal Klang

**Affiliations:** 1 BRIDGE GenAI Lab, Beth Israel Deaconess Medical Center, Harvard Medical School, Boston, Massachusetts, United States of America; 2 Department of Neurology, Beth Israel Deaconess Medical Center, Harvard Medical School, Boston, Massachusetts, United States of America; 3 The Windreich Department of Artificial Intelligence and Human Health, Mount Sinai Medical Center, New York, New York, United States of America; 4 The Hasso Plattner Institute for Digital Health at Mount Sinai, Mount Sinai Health System, New York, New York, United States of America; 5 Department of Radiology, Beth Israel Deaconess Medical Center, Harvard Medical School, Boston, Massachusetts, United States of America; 6 The Charles Bronfman Institute for Personalized Medicine at Mount Sinai, Icahn School of Medicine at Mount Sinai, New York City, New York, United States of America; National Tsing Hua University, TAIWAN

## Abstract

Clinical AI systems can produce different recommendations when a patient’s race, income, housing status, or gender identity changes, but a differential alone does not establish unjustified bias. What separates a clinically justified differential from an unjustified one is not its size or direction but its origin: whether the feature acts through a clinically justified pathway, or marks how patients of that group have been treated. That distinction is causal, and a model trained only to detect statistical association records that a feature carries information but never why. Existing evidence does not establish reliable recovery of this adjudication inside that model; we therefore argue for explicit causal and normative supervision, whether supplied by an external layer, built into training, or both. We set out a six-criterion rubric, four approaches to implementing it, and a two-sided evaluation reporting preserved clinical sensitivity alongside reduced bias.

## Introduction

Clinical AI systems are entering clinical workflows despite evaluations documenting clinically consequential biases and emphasizing the need for comprehensive bias assessment before integration [1]. Large language models (LLMs) are a prominent recent example; our previous work documented substantial sociodemographic differences in their clinical recommendations, read as bias [2].

We propose a complementary interpretation: these differences vary in origin, and origin is what governance must track. They are not simply defects to eliminate but signatures of the same mechanism that makes these models clinically useful: context-sensitive pattern recognition.

## What we mean by bias

Bias here means systematic over-association: differential recommendations for otherwise identical patients, beyond clinical justification. The difficulty sits in that last clause. Ancestry, for which Black race is an imperfect proxy, raising the prior for sickle cell disease in a patient with acute chest syndrome, prompting confirmatory testing, reflects a heritable trait’s distribution; a higher pain-treatment threshold reflects false beliefs clinicians have been documented to hold [3,4]. Both register in an audit as race sensitivity; what separates them is origin, and the observed differential alone does not establish which origin produced it.

## Bias and capability share a mechanism

Any clinical model fit to data conditions its output on correlated contextual signals; attention-based language models [5] do so across an entire note, the capability that makes them clinically useful. Training data are marked by historical inequity, and no corpus of language-model scale can be audited clause by clause for the mechanism behind every association, so curation reduces the problem rather than resolving it [6].

Mitigation’s capability cost depends on the feature and method: targeted techniques (concept-space projection [7,8], counterfactual data augmentation [9], and preference optimization, a general steering method adaptable to a specified behavior [10]) have downstream performance effects, if any, varying by method, task, and evaluation. We do not claim a general tax on mitigation; the cost is clearest when a model is made invariant to a feature that predicts the outcome through a clinical mechanism, and that information is simply gone.

## The distinction is causal, not statistical

Table 1 sorts contextual features into legitimate and illegitimate uses by *why* the feature carries information, not by an audit’s usual sorting principle. Sickle cell is more common in Black patients through inheritance of hemoglobin S: race marks an ancestral distribution; the mechanism is the genotype, which criterion 3 asks to obtain rather than assume. Black patients receive less analgesia because of what clinicians believe about them, a differential mediated by discriminatory belief and treatment rather than physiology [4]. Only one would survive a world where the injustice had been removed.

**Table 1:** Legitimate contextualization and illegitimate bias, sorted by causal structure. In most rows, both columns describe a differential response to the same variable, and both would appear in an audit as sensitivity to it. The race and ethnicity, sexual orientation, and gender identity rows are the instructive exceptions: their legitimate column replaces the feature with a more proximal variable (ancestry or genetic testing, reported behavior, organ inventory), which is what criterion 3 of Table 2 asks for. What separates them is the fourth column: whether the feature acts through a clinically justified pathway, or primarily marks how patients carrying it have been treated. Discriminatory treatment is itself a causal route to the association, just not one that justifies acting on it. Sex is used for physiological claims and gender identity for social and identity claims; the table separates them because they are not interchangeable. Race and ethnicity are used throughout as a single compound social category, not as a biological variable in its own right.

Contextual feature	Legitimate contextualization	Illegitimate bias	Causal question
Age	Adjusting drug dosing for pediatric or geriatric pharmacokinetics	Deprioritizing older patients for interventions of proven benefit	Does age alter drug handling, or stand in for a judgment about whose treatment is worth funding?
Sex	Recognizing that women with acute coronary syndromes more often also report dyspnea or nausea alongside chest pain, and adjusting the workup accordingly	Attributing a female patient’s reported pain to psychological causes	Does sex alter the pathophysiology, or how the report is received?
Race and ethnicity	Using reported ancestry, family history, or hemoglobinopathy testing to evaluate for sickle cell disease in a patient with acute chest syndrome; race alone may prompt the workup but should not substitute for it	Applying a higher threshold before treating a Black patient’s pain	Does the proximal marker carry a heritable distribution, or does race stand in for what clinicians have believed?
Housing status	Coordinating discharge planning and follow-up for an unhoused patient	Recommending psychiatric admission for an unhoused patient presenting with chest pain	Does it identify a resource the patient lacks, or a suspicion attached to the patient?
Income and insurance	Discussing medication cost and adherence barriers with the patient	Ordering fewer diagnostic tests for an uninsured patient	Does it identify a barrier to be addressed, or a constraint applied to the patient?
Sexual orientation	Assessing sexually transmitted infection risk from behaviors this patient has reported	Inferring risk from orientation alone, without asking about behavior	Is the risk carried by a reported behavior, or an assumption about behavior?
Gender identity	Recognizing that organ inventory rather than recorded gender determines which screening applies	Presumptive mental health referral for transgender patients regardless of presentation	Does the feature stand in for anatomy, or is the identity itself treated as the finding?

That yields a usable test, related to but distinct from counterfactual fairness’s causal-model intervention on the protected attribute itself: [11] would the association persist if discriminatory care and unsupported group assumptions were removed but the legitimate pathway held? Sickle cell would; the analgesia gap would not: two causal structures leaving the same statistical signature, not two regions of one spectrum.

## Why the model is unlikely to draw the line unaided

This is a distinction no purely predictive training objective is built to certify: parameters are optimized to reduce predictive loss, recording that a feature carries information, never why: two features of identical predictive value and opposite moral standing are, to the objective, the same feature. A benchmark testing causal inference in language models finds performance near-chance [12], and apparent competence may reflect recalled claims rather than reasoning about the case [13]. Mitigation is not futile, but it cannot reliably supervise itself: something external must say which feature, in which context.

## The problem is older than transformers

Nothing here is specific to attention: any model fit to observational health data absorbs the regularities it contains, including unequal care. Race coefficients were written by hand into kidney- and lung-function equations before any were learned, and removing them required a causal argument, not a fairness metric [14,15]. The most consequential documented case used no demographic variable: a risk score for millions used health *costs* as a stand-in for health *needs*, and because less had been spent on Black patients at a given illness level, it understated their need; no feature-list inspection would have flagged it [16]. Language models change the surface, not the kind, by reading the whole note and widening the aperture for both reasoning and inherited prejudice.

## What we are and are not arguing

The prevailing position is not that models should be blind to context: justified context should be preserved while unjustified differentials are removed [17], a goal we share. Our disagreement is about the instrument: enforcing feature invariance without adjudicating its causal role answers a causal question with a statistical procedure, removing both kinds of sensitivity because it cannot tell them apart. If the criterion is causal and the model is associative, existing evidence does not establish reliable recovery from training data alone; we therefore propose explicit causal and normative supervision: an external layer, built into training, or both. Models with differentials of undocumented origin are not ready for autonomous clinical use [1,2], and the rubric, layer, evaluation, and audit we propose address that deficit without being sufficient alone.

## A working rubric

Table 2 sets out six questions, offered for criticism and not summed, to answer on the record before a contextual feature may influence a recommendation; summing would flatten causal questions into one number. Criteria 1–3 turn on mechanism and causal structure and sit with a standing multidisciplinary panel (clinicians, causal-inference and epidemiology expertise, and local implementation leadership, conflicts disclosed) rather than no named owner. Criterion 5 (anchoring) belongs to specialty societies; criteria 4 and 6 concern harms experienced rather than measured and require patient representation. Where criteria conflict, the system surfaces the influence to the clinician rather than acting on or suppressing it, with dissent recorded alongside the rule.

**Table 2:** A working rubric for adjudicating whether a contextual feature may influence a clinical recommendation. Applied to a specific feature, in a specific context, for a specific decision. The criteria are not weighted or summed; each is a question that must have an answer on the record.

#	Criterion	The question	Fails when
1	Mechanism	Can a mechanism (physiology, pharmacokinetics, heritable variation, exposure, transmission risk, or a concrete resource need) be named through which this feature affects this outcome?	The only justification is that the association is in the data. Predictive strength is not a mechanism.
2	Counterfactual survival	Would the association persist in a world where discriminatory care and unsupported assumptions about the group had been eliminated but the legitimate pathway was unchanged?	The association exists because of how patients of this group have been treated, not because of a pathway that would survive that removal.
3	Substitutability	Is there a more proximal variable (an ancestry-informative marker, a laboratory value, a reported behavior, a documented need) carrying the same information?	A measurable proximal variable exists, is available at the point of decision, and this feature (a demographic label or any other proxy, such as cost standing in for clinical need) is being used in its place. Where the proximal variable is not yet available, the feature may raise the prior and prompt obtaining it, but should not substitute for it.
4	Specificity	Where the mechanism named under criterion 1 operates through an individual-level finding (a symptom, behavior, stated concern, or result) rather than the feature acting on the patient directly, has that finding been obtained?	The mechanism requires an individual-level finding, and the recommendation acts on group membership before that finding is obtained. Does not apply where an established clinical rule legitimately conditions directly on the feature under a criterion-1 mechanism and no separate patient-specific mediator is required, as with age-defined dosing guidance.
5	Anchoring	Is the differential recommended by a current specialty guideline, or supported by an epidemiological estimate with a citable source and stated source population?	The differential is modeled on observed practice rather than a stated standard.
6	Direction and asymmetry of harm	Does incorporating the feature expand what is offered, or withhold, ration, or redirect? What is the cost of being wrong each way?	Acting on the feature expands or restricts care without evidence proportionate to the greater expected harm of being wrong in either direction.

## Population risk is not a patient finding

We found LGBTQIA+-labeled patients routed to mental health evaluation for presentations with no psychiatric content [2]. Epidemiology is not in dispute: depression and suicidality are elevated among lesbian, gay, and bisexual people through discrimination falling unevenly on individuals [18], but a population prior is not a patient finding. Criterion 4 of Table 2 is why: demographic context may adjust a prior but may not substitute for an individual-level finding the mechanism requires. No referral should follow from a group label without a patient-specific finding; the recommendation is instead revised or escalated for review, never executed on the label alone; the same rule applies to a presumptive psychiatric admission for an unhoused patient with chest pain.

## Context about context: The missing metacognitive layer

Clinical AI systems need context about their context: a layer governing when associations should influence a decision. Four instruments follow, each implementing the rubric of Table 2.

First, **counterfactual supervision.** Two constructions, not one: a *controlled pair* holds every variable but the descriptor fixed, testing for unjustified sensitivity; a *causally coherent counterfactual* lets the descriptor’s legitimate descendants change with it under an explicit causal model, testing whether the justified pathway survives. Either way, the response is fixed by the rubric, not observed practice, with a nameable mechanism required for any permitted change or no differential by default. Evaluation needs two numbers: invariance where the rubric says match, sensitivity where it says not; reducing both is flattening, not debiasing.

Second, **guideline retrieval from a normatively specified teacher**: rubric-derived rules and an adjudicated case bank, each naming its criterion, queried at inference and versioned by mechanism, source, and source population; a guideline revision updates the rule set without retraining, though a latency-constrained deployment can distill it into a compact model, re-distilled on each revision [19].

Third, **agentic oversight** (Fig 1): a reasoning agent emits decision-relevant features; audit, guideline, and specificity agents apply criteria 1–3, 5, and 4; an arbiter applies criterion 6 and passes, revises, or surfaces to the clinician, with no branch executing autonomously or suppressing from view. This is a specification, not a reported system; the pattern follows agentic clinical systems reviewed elsewhere [20]. Over-conservatism is tracked the same way: the rate these agents revise or escalate a rubric-compliant differential, alongside the bias-reduction rate.

**Fig 1:**
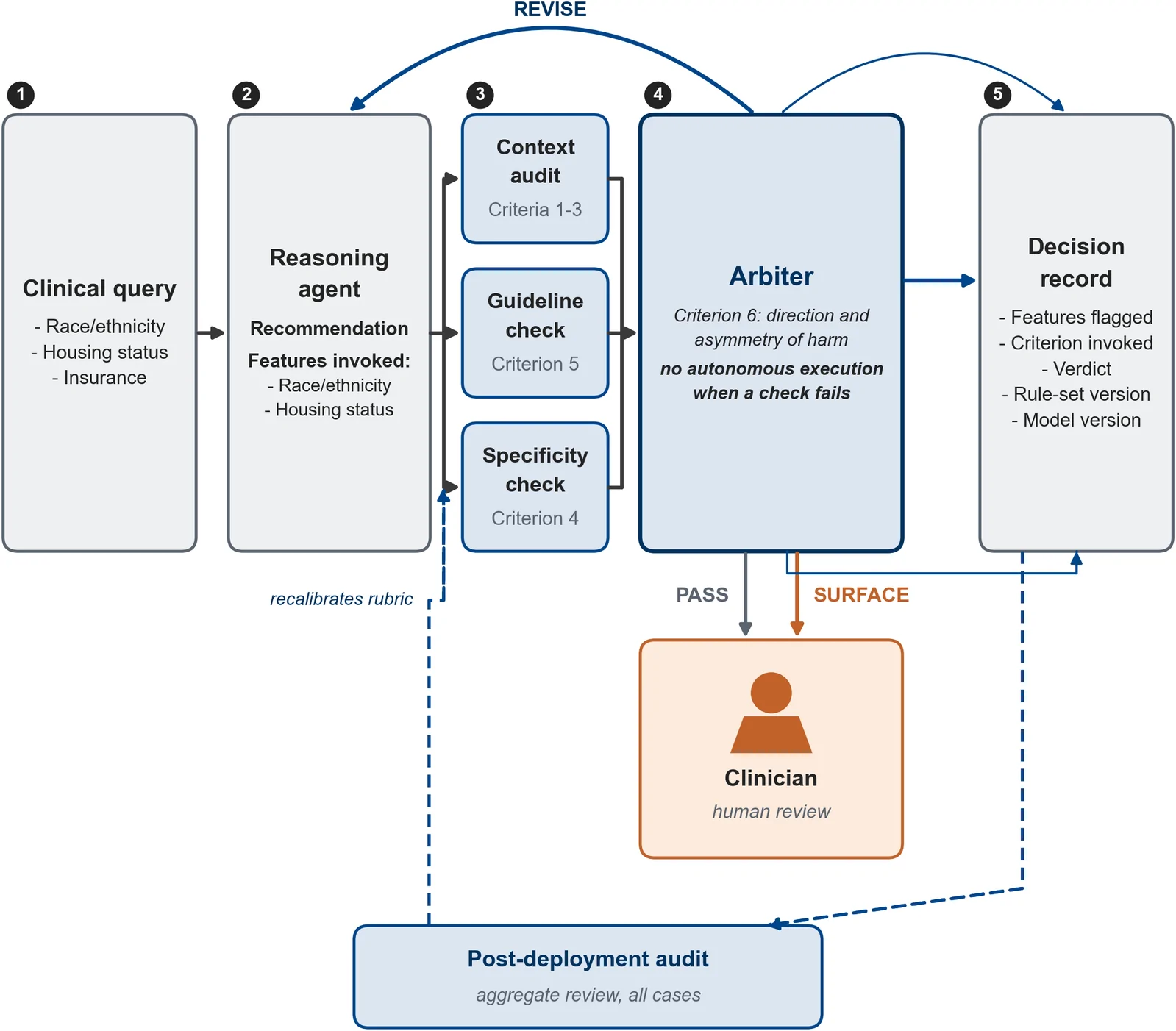
A minimal architecture for adjudicating contextual influence. A clinical query carrying contextual features (1) reaches a reasoning agent, which returns a recommendation together with a list of the features it treated as decision-relevant (2). That list is what everything downstream operates on, and it is not accepted at face value: a self-reported list can be incomplete or a post-hoc rationalization, so it needs independent verification (counterfactual perturbation, attribution methods, or external audit), and a model that cannot produce a list amenable to that check cannot be governed this way. Three checks then apply the rubric of Table 2 (3): a context audit applies criteria 1 to 3, a guideline check applies criterion 5 (anchoring), and a specificity check applies criterion 4. An arbiter (4) applies criterion 6 and has three actions and no fourth: pass the recommendation to the clinician as routine output, return it for revision, or surface it to the clinician with the disagreement stated. None of the three actions executes a failing recommendation autonomously.

The architecture has no silent-suppression pathway: a recommendation that fails a check is never discarded from the record, and none is autonomously executed. Each is instead returned for revision or surfaced to the clinician with the disagreement, uncertainty, and required level of review stated. Surfacing carries its own risk: anchoring and automation bias can lead a clinician to over-weight a flagged suggestion even when it is wrong, which is why the record in (5) keeps what was surfaced and why, open to audit rather than treated as resolved by disclosure alone.

All three actions write to the decision record (5): the features flagged, the criterion invoked, the verdict, and the rule-set and model versions. The pass path is drawn straight into the record for legibility; the revise and surface paths write the same record through their own connectors, and revised and surfaced cases are the ones an audit examines most closely. Those records are what post-deployment audit aggregates, and the dashed return path shows what that audit revises: not the model, but the versioned rule set the three checks apply, so a changed guideline or a reclassified feature propagates without retraining.

Fourth, **continuing audit after deployment.** None of the above survives contact with time: guidelines change, case mix shifts, and a feature’s classification is revisable [14,15]. Deployed systems need scheduled counterfactual probing, monitoring for proxy drift in any feature (the kind the cost-based score showed [16]), override logging, and a body empowered to retire a rule [21].

## Scope and limitations of this argument

This conceptual paper adds no experiments; the claims above are propositions, not findings. The strongest, that mechanism and history are unlikely to be told apart inside an associative model, is testable: a model sorting associations by causal structure and generalizing to unseen labels would weaken it substantially. Recent evaluations are consistent with this concern: 2026 assessments of clinical and real-world causal reasoning report meaningful but still incomplete performance [22,23]. Their distance from the near-chance benchmark above [12] reflects task formulation (formal inference from stated correlations versus reasoning over contextualized clinical and real-world text); neither result supports the reliable, generalizable adjudication the argument requires. That suppression costs capability is argued, not measured; the paired metrics above specify how. Governance is one promising route, not the only one: retrieval-constrained systems, narrow task-specific tools, and structured-input pipelines also shrink the surface for inherited prejudice, at the cost of the contextual integration a general model provides. Related work on path-specific counterfactual fairness distinguishes permissible from impermissible causal pathways [24]. Our contribution is the clinical governance problem of specifying those pathways and determining whether a model’s contextual influence is justified in a given decision.

## Where this leaves clinical deployment

Clinical AI can benefit from contextual information, but the data from which these systems learn encode both disease biology and historical patterns of how patients were treated. Current evidence does not establish that the model itself can reliably separate the two: no reason to withhold or flatten them, but a specification for what to build alongside them (a stated rubric, a layer applying it, an evaluation reporting what was preserved and removed, and an audit that keeps asking).
